# Real world data of Ewing sarcoma from a resource-limited setting with poor compliance to treatment leading to poor outcomes

**DOI:** 10.3332/ecancer.2024.1801

**Published:** 2024-11-14

**Authors:** Nidhi Gupta, Kislay Dimri, Sudhir Kumar Garg, Aanchal Arora, Awadhesh Kumar Pandey

**Affiliations:** 1 Department of Radiation Oncology, Government Medical College and Hospital, Chandigarh 160030, India; 2 Department of Orthopaedics, Government Medical College and Hospital, Chandigarh 160030, India; 3 Department of Community Medicine and School of Public Health, Post Graduate Institute of Medical Education and Research (PGIMER), Chandigarh 160012, India; a https://orcid.org/00 00000340792631

**Keywords:** Ewing, sarcoma, resource-limited setting, compliance, survival

## Abstract

**Background:**

There is limited data from India on Ewing sarcoma (ES) patients. We analysed the demographic and clinical profile of ES patients, the systemic chemotherapy, local treatment and outcomes in patients with localised, metastatic and recurrent disease.

**Methods:**

Data of ES patients reporting from 2010 to 2019 to a tertiary care referral centre in north India was evaluated. A total of 81 patients were retrieved of whom 76 were assessed for treatment and outcomes. Patients were stratified as per localised (LD) or metastatic disease (MD). Outcomes were evaluated in terms of 3-year and 5-year disease-free survival (DFS) and overall survival (OS). Prognostic factors influencing OS for patients with LD were assessed.

**Results:**

The majority (68%, n = 55) of patients presented from rural areas with 30% (n = 24) presenting 6 months after the onset of symptoms, 63% (*n* = 51) had primary tumours more than 8 cm and 7% (*n* = 6) had a pathological fracture at presentation, 78% (*n* = 63) patients had LD while 22% (*n* = 18) patients had MD. Local treatment consisted of surgery in 56% (*n* = 28) patients and definitive radiotherapy in 44% (*n* = 22) patients. Compliance with chemotherapy was poor with patients receiving a median of five and seven cycles of chemotherapy as neoadjuvant and consolidation chemotherapy, respectively. Three-year OS for LD, MD and overall cohort was 41%, 6% and 32%, respectively. Size of the primary tumour > 8 cm, completion of less than 15 cycles of chemotherapy and presence of MD was associated with inferior survival on multivariate analysis.

**Conclusion:**

Poor outcomes were reported in this cohort of ES patients from a resource-limited setting where patients have a delayed diagnosis, advanced disease and poor compliance to adjuvant consolidation chemotherapy secondary to geographical, social and financial barriers. There is an urgent need to address these barriers for low middle-income countries to improve outcomes.

## Introduction

Ewing sarcoma (ES) is the second most common primary bone cancer after osteosarcoma. It occurs predominantly in children and adolescents. It is a rare disease and makes up 1% of childhood cancers in children and adolescents [1, 2]. The incidence of ES in the United States was 3.93 per million during the period between 1973 and 2004 [3]. According to an Indian registry, bone cancers represented 0.9% of all cancers with ES as the most common bone cancer [4]. As per another hospital-based cancer registry (HBCR) from India osteosarcoma was found to be the most common bone sarcoma, followed by ES [5].

Reciprocal translocation *t* (11;22)(q24;q12), resulting in *EWRS1-FLI1* fusion, can be detected in 85% of cases of ES, whereas 20% of cases of ES harbour the (21; 22)(q22; q12) translocation [6]. ES can also arise from the soft tissues, besides the bone and most commonly involves the long bones, pelvis, chest wall and spine [6]. The most common presenting symptoms include pain and swelling. ES usually presents as localised disease (LD) with 25% of cases presenting as metastatic disease (MD) [7].

Treatment of ES involves a multimodality approach with the intent to provide the best outcomes and a good quality of life in these young patients [8, 9]. Treatment has evolved over time with the incorporation of multiagent chemotherapy with interval compressed schedules. Local treatment includes surgery or radiotherapy or a combination of both. Local treatment is followed by a prolonged consolidation schedule lasting for a period of 6–9 months [6, 8]. Even with advances in treatment for ES, outcomes remain poor with 5-year survival of 60%–75% in LD and 20%–40% in MD [6–8].

Treatment of ES is resource-intensive and requires a dedicated multidisciplinary team, infrastructure and finances, which is a challenge for a resource-limited country like India. In India, there are limited oncology centres dedicated to the management of ES, with corresponding minimal literature available on epidemiology, clinical characteristics, treatment protocols, outcomes and prognostic factors of Indian patients. The majority of patients present with advanced disease secondary to delayed diagnosis [10]. Compliance to treatment is poor leading to poor survival outcomes [11].

We aim to present the real-world experience of managing ES in a tertiary care referral centre in North India, where compliance to treatment is poor and outcomes are significantly inferior than that reported as per Western and other Indian studies.

## Material and methods

This is a hospital-based study that was conducted at the Department of Radiation Oncology of a tertiary care hospital in north India. HBCR is maintained in the Department of Radiation Oncology. The HBCR data on all ES patients were retrieved for a period of 10 years from 2010 to 2019. A total of 81 patients of ES were identified from the retrospective records. All 81 patients were evaluated for demographic and clinical profile while 76 patients were evaluated for treatment and outcomes after excluding patients who did not report for treatment.

Only histopathologically proven patients of ES were included in the analysis. Data were analysed for demographic profile including age at presentation, gender, rural or urban residence, any pre-existing morbidities or addiction. Clinical profile was evaluated for symptoms at presentation, duration of symptoms before initiating treatment, site, laterality, the radiological investigation done, maximum size of the primary tumour and presence of LD or MD.

Treatment for LD or for patients with curative intent consisted of delivering 4–5 cycles of neoadjuvant chemotherapy (NACT) followed by local therapy which included surgery or radiotherapy or both, which was followed by adjuvant consolidation chemotherapy to complete 1 year of therapy [12, 13]. The details of neoadjuvant and adjuvant chemotherapy delivered in terms of regimen, the number of cycles, toxicity and timing with respect to local treatment were analysed. Surgery and radiation details were evaluated.

Recurrence patterns, treatment for recurrent and MD were also analysed. Outcomes were evaluated in terms of disease-free survival (DFS) and overall survival (OS). OS was calculated from the date of registration in the department to till death or the last follow up while progression-free survival (PFS) was calculated from the date of registration to the first event (local recurrence, metastases or death). Prognostic factors affecting OS for patients with LD were assessed.

As per our institutional protocol, 4–5 cycles of NACT consisting of alternating Vincristine/Adriamycin/Cyclophosphamide (VAC) and IE cycles were delivered every 3 weeks, consisting of [14].

Cycle 1-Day 1: Vincristine 1.5 mg/m^2^ (max 2 mg) IV, Doxorubicin 75 mg/m^2^ IV or Dactinomycin 1,250 mg/m^2^ IV (to be substituted for doxorubicin when cumulative lifetime doxorubicin dose of 375 mg/m^2^ has been delivered) and Cyclophosphamide 1,200 mg/m^2^ IV with Mesna support.

Cycle 2-Days 1–5: Ifosfamide 1,800 mg/m^2^ IV with Mesna support (60% of ifosfamide dose), Etoposide 100 mg/m^2^ IV.

Following local therapy, the chemotherapy schedule was continued with the aim of completing 17 cycles. Response to NACT was assessed clinically and radiologically and informed decision was taken between surgery and/ or radiotherapy. Postoperative radiotherapy (PORT) after surgery was delivered for margin-positive disease. Preoperative radiotherapy was delivered for bulky disease with either progressive disease or no significant downstaging of disease after NACT, in an attempt to make the disease resectable for R0 resection or limb salvage surgery. Definitive radiotherapy was delivered if R0 resection was not feasible or if the family refused surgery. Management for patients with recurrent or MD was individualised based on disease burden, site of metastases, general condition of patient and family decision.

Statistical analysis was done using Statistical Package for Social Sciences version 17 (Chicago, IL, USA). Descriptive statistics were used for demographic, clinical parameters and treatment modalities. OS and PFS were estimated according to the Kaplan–Meier method, stratified by the LD and MD. Univariate and multivariate (Cox proportional hazards regression model) analyses were used to assess the factors influencing OS in patients with LD. Age of patient (>15 years), duration of presenting symptoms (>6 months), primary site (pelvis versus extremity), primary tumour size (>8 cm), presence of pathological fracture, number of chemotherapy cycles, use of surgery as local treatment were included as covariates on univariate and multivariate analysis.

A waiver from the Institutional Ethics committee was taken for the study as this study did not involve patient interaction or intervention.

## Results

### Demography

The median age at presentation was 16 years with 48% (*n* = 39) of patients belonging to the 11–20 years age group. There was a male preponderance with a male-to-female ratio of 1.7: 1. In our registry, 68% (*n* = 55) of patients were from a rural background. In this young population, 80% (65) patients were single, 91% (*n* = 74) patients had no co-morbidities and 88% (71) patients had no addiction (Table 1).

### Clinicopathological profile

Pain and swelling were the most common presenting symptoms reported by more than 70% of patients, restriction of movement was reported by 24% (*n* = 19) patients and 12% (*n* = 10) patients gave a history of trauma. The majority of patients (44%, *n* = 36) had a late presentation, 4–6 months after the onset of symptoms and 63% (*n* = 51) patients had a primary tumour >8 cm at presentation with 7% (*n* = 6) patients presenting with a pathological fracture. The most common sites of presentation were extremities (64%, *n* = 52) followed by pelvis (24%, *n* = 19), soft tissue (7%, *n* = 6) and axial skelton (5%, *n* = 4). The femur and tibia were the most common long bones affected. Conventional radiographs were done for all patients at presentation. Magnetic resonance imaging (MRI) was the most common (90%, *n* = 73) investigation for the primary while computed tomography (CT) chest was most commonly used to rule out MD. However, the diagnosis of ES on radiology was consistent only in 43% (*n* = 35) patients and the confirmation of the final diagnosis was obtained on biopsy. The most common positive immunohistochemistry markers were CD99, PAS, FLI1, Ki 67, chromogranin and NSE. Bone marrow was positive in 9% (*n* = 7) patients. Overall, 78% (*n* = 63) patients had LD at presentation and 22% (*n* = 18) had MD (Table 2).

### Systemic treatment

All patients with LD received NACT with a median of 5 cycles. In the neoadjuvant setting, VAC/IE regimen was used in 91% (*n* = 53) patients while the rest received VAC regimen only due to poor general condition. Local therapy was followed by adjuvant chemotherapy with the aim to complete a total of 17 cycles; however, a median of 7 cycles only were received in the adjuvant setting, where 95% (*n* = 39) patients received VAC/IE regimen while 5% (*n* = 2) patients received VAC only (Table 3). Compliance with adjuvant chemotherapy was poor, with 24% (*n* = 10) of patients defaulting to adjuvant chemotherapy. During or within 4 weeks after completing adjuvant chemotherapy, 39% (*n* = 16) of patients had progressive disease.

### Local treatment

Local treatment included either surgery or local radiotherapy or both. Eighty six percent (*n* = 50) patients received some form of local therapy, 56% (*n* = 28) patients underwent surgery while another 44% (*n* = 22) underwent definitive radiotherapy. Amongst the patients undergoing surgery, 22 patients underwent limb salvage surgery. The majority of patients with tumours in the pelvis or axial skelton received definitive radiotherapy. Patients with positive margins and another patient with skin infiltration received postoperative radiation (*n* = 4) after surgery. Preoperative radiation was received by six patients. Details of radiation dose are listed in Table 3.

### Treatment for relapse, progressive or MD

The most common sites of recurrence as well as metastases at presentation were in the lungs followed by bones. Chemotherapy followed by radiotherapy were the common treatment modalities used for these patients. The details of treatment are reported in Table 4.

### Outcomes

**Figure 1.**
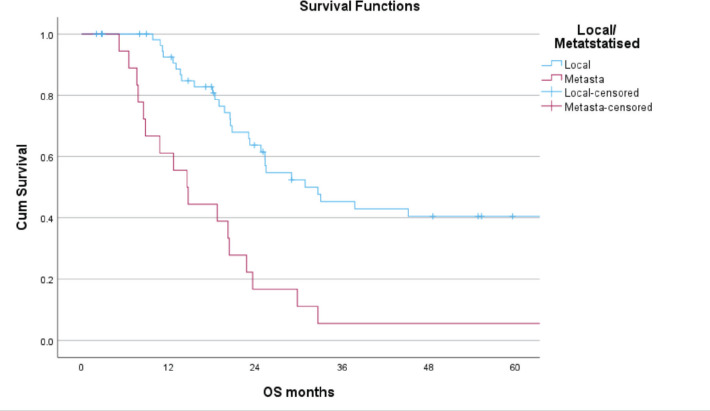
Five-year OS of patients with local and metastatic ES.

The median follow up was 20.4 (0–161) months. The 3-year OS for patients with LD, MD and the entire cohort was 41%, 6% and 32%, respectively, while the 5-year OS for patients with LD was 35% and none of the patients with MD survived beyond 4 years (Figure 1). The 3 and 5 years DFS for patients with LD was 56% and 15%, respectively. The size of the primary tumour <8 cm and number of chemotherapy cycles received > 15 in patients with LD were found to be statistically significant for improved OS on both univariate and multivariate analysis (Table 5).

## Discussion

Our analysis presents real-world data from a resource-limited country where outcomes are significantly inferior and greatly influenced by delayed diagnosis, advanced presentation, social, financial and geographical barriers leading to poor compliance and poor outcomes to treatment [9, 11, 15]. 

### Sociodemographics

The median age at presentation, male preponderance, most common symptoms, most common sites of presentation and stratification as per LD or MD in our analysis are similar to global and national statistics [6, 8, 16, 17].

Our patients show a delayed presentation and report with advanced disease. The average duration of symptoms as per literature is 3 months [6, 10] while 44% (*n* = 36) of our patients and another 30% (*n* = 24) present within 4–6 months and more than 6 months of symptoms, respectively. Nearly 63% (*n* = 51) of patients in our study present with a primary tumour >8 cm as opposed to other Indian studies [16] with a smaller primary. The incidence of pathological fracture in our study (7%, *n* = 6) is similar to that reported in other Indian studies (6%) [18] signifying advanced disease, further 12% (*n* = 10) patients give a history of trauma drawing their attention to the pathology. Nearly 70% (*n* = 55) of patients in our study report from rural areas, where unsuspecting primary care clinicians, coupled with a history of trauma, nonspecific clinical symptoms and lack of advanced diagnostic facilities, fail to diagnose and refer the patients in timely to tertiary care centres [10]. In a sub-group analysis for patients above 30 years, 5 patients were identified whose clinical characteristics matched with that reported by another study from India and included patients with the extra-skelteal disease who had poor outcomes [19].

### Management

The majority of our patients underwent MRI for the primary and CT chest for metastatic workup while PET CT was done in less than 5% (*n* = 4) patients signifying the financial constraints [15, 20]. Fertility counselling was done for all patients but none of them opted for it due to financial constraints [15, 21]. Multiagent NACT for 9–12 weeks prior to local treatment helps to downstage the disease and increase the probability of R0 resection and facilitate limb salvage surgery. The addition of adjuvant consolidation chemotherapy for an overall treatment duration of 6–9 months further helps to improve outcomes [6, 8, 12]. The current standard of care for chemotherapy is either the EURO-EWING99 trial [22], utilising a dose-intense chemotherapy approach with four-drug combination vincristine, ifosfamide, doxorubicin, etoposide (VIDE) during induction and VAI or VAC consolidation (vincristine, actinomycin D, ifosfamide/cyclophosphamide), or the AEWS0031 trial by COG [23] that uses the dose-dense approach VDC/IE regimen (vincristine, doxorubicin, cyclophosphamide/ifosfamide, etoposide) utilising interval-compressed cycles of chemotherapy administered 2-weekly rather than 3-weekly. The interval-compressed VDC/IE regimen showed superiority to VIDE for both event-free survival (61% versus 55%) and OS (72% versus 64%), with similar toxicity, and it is currently the preferred first-line treatment in ES [24]. All studies on ES from India have used non-dose-dense chemotherapy [17, 18, 25]. VAC alternating with IE given every 3 weeks is the regimen used in our analysis as per our Institutional protocol. Haematological toxicity with chemotherapy included anaemia and neutropenia is 9% (*n* = 5) and 21% (*n* = 12), respectively. One patient died of septic shock secondary to neutropenic sepsis.

In our study, nearly 70% (*n* = 55) of patients reported to tertiary care centres from rural areas, who after local treatment show poor compliance to consolidation chemotherapy, especially when they are explained about the prolonged treatment duration lasting over a year, finances and subsequent follow up. The median number of chemotherapy cycles received are 5 and 7 prior to and after local treatment. This is due to several reasons including long distance from their home, logistics of travelling, stay, work loss, language barriers, chemotherapy induced toxicities, risk of infertility, financial constraints and the belief that disease has been taken care of after local treatment. The use of dose-dense chemotherapy for patients with localised ES in real world setting for low middle income countries (LMICs) should be evaluated, keeping in mind poor tolerance, poor supportive care, cost and additional toxicities that could further decrease the compliance and outcomes with chemotherapy [17].

Local treatment is planned in a multidisciplinary meeting 9–12 weeks after NACT with clinical and radiological response assessment. Delay beyond 16 weeks negatively impacts outcomes [6, 26]. Local treatment may consist of surgery or radiotherapy depending upon the extent of residual tumour, morbidity resulting from resection or radiotherapy, patient and family preferences. Literature, unanimously reports that the results of surgery for all sites are better than those for radiotherapy. Local recurrence rates with radiotherapy are reported to be 30% versus 10% with surgery [27]. There is no role for debulking surgery in ES, so surgery should be attempted if R0 resection can be achieved. Pelvic tumours which cross the midline, involve major viscera or require pelvic organ removal, may not be considered for surgery [6, 8]. Quality of life is especially important for childhood malignancies where the aim is to provide a cure with function preservation [9, 28]. Limb salvage surgery becomes the preferred treatment. In our study, 22/28 of patients in the surgery arm had undergone limb salvage surgery. Amputation is considered when negative margins cannot be achieved without compromising the functional outcomes.

Preoperative radiotherapy (45–54 Gy) is usually considered for large tumour volumes where close or positive resection margins are expected postoperatively or to facilitate limb preservation surgery [30, 31]. In our analysis, 19% (*n* = 6) patients received preoperative RT after poor response to NACT. Patients receiving definitive radiotherapy as local treatment are those with advanced, axial/ pelvic tumours, bulky disease or those who have responded poorly to NACT and hence likely to have poorer outcomes compared to patients undergoing surgery [27, 31]. In LMICs, where patients present with advanced disease and access to specialised surgeons is limited, radiotherapy becomes a dominant modality of treatment. In our analysis, 28 and 22 patients underwent surgery and radiotherapy (50–60 Gy) as definitive local treatment, respectively. In a study from South India, surgery and definitive radiotherapy were given to 59.5% and 28% of patients, respectively [16]. Patients undergoing local treatment, often refuse surgery secondary to cultural beliefs, social stigma and financial constraints limiting endoprosthesis accessibility.

Benefits and indications for PORT remain controversial, with the only universal consensus being positive or close margins (>2 mm) [29, 31]. Other relative indications for which PORT has been used include, low percentage necrosis after NACT, large preoperative soft tissue component, large tumour volume > 300 cc, pathological fracture, skin tumours with pleural effusion, non-sacral pelvic ES or spinal and paraspinal disease where wide surgical margins are unlikely [6, 8, 27, 29]. PORT (45–60 Gy) at our institute is added for positive margins, and one of the patients received it following limb salvage surgery where at a diagnosis the skin was involved by tumour.

Treatment of patients with extra-skeletal ES (7%) in our analysis included multiagent chemotherapy and radiotherapy in the majority, based on the principles of skeletal ES [6, 8, 14].

Recurrent ES, has poor outcomes with distant metastases being more common than local recurrences as seen in our study [7]. In a sub group analysis of 39% (*n* = 16) patients who progressed either on treatment or within 4 weeks of treatment, the median size of the tumour at presentation was 12 cm (5–26) against the median size of the cohort which was 9.5 cm (2–26 cm). Chemotherapy is the main modality of management and may include alkylating agents, topoisomerase inhibitors, irinotecan, temozolomide, gemcitabine, docetaxel and so on, used alone or in combination. Radiotherapy may be preferred for local treatment of primary or oligometastatic sites [6, 8, 14].

Patients with metastases at diagnosis are treated based on the disease burden. Patients with extensive metastases are taken for palliative treatment and chemotherapy with VAC only may be preferred to limit the toxicities [6, 7]. Patients with oligometastases can be treated on lines of LD with NACT followed by local therapy and additional radiotherapy for oligometastases, followed by consolidation systemic therapy [6, 7, 14]. In our analysis, 60% (*n* = 11/18) of patients with MD were treated with VAC/IE. Whole-lung irradiation may be considered, in patients with good response [6, 7]. The choice of regimen in second-line therapy should be based on patient profile and drugs used previously [7,8].

### Outcomes

The outcomes in our study were significantly inferior to those reported by various Indian studies and Western literature. The 3 year OS for patients with LD, MD and the entire cohort was 41%, 6% and 32%, respectively, while the 5 year OS for patients with LD was 35%. The 3 and 5 years DFS for patients with LD was 56% and 15%, respectively. In a study from South India, 5 year OS for LD, MD and overall cohort was 57.2%, 4% and 43.7%, respectively, while the 5 year EFS for LD was 56.6% [16]. In another study by Biswas *et al* [25] 5-year EFS and OS of 36.8% and 52.4% were reported in a cohort of 224 patients with localised ES. In a study from TMH India, 3 year OS for LD, MD and overall cohort was 82.8%, 65.3% and 79.3%, respectively, in a population of adolescents and adults with ES [18].

On univariate and multivariate analysis the factors that were statistically associated with poor survival in patients with LD included presence of MD at diagnosis, size of the primary tumour >8 cm and total number of chemotherapy cycles received. Patients receiving <15 cycles of chemotherapy had inferior survival compared to those who received 15 or more cycles, this can be understood as the cumulative dose of chemotherapy decreases.

The poor outcomes in our study arise from various geographical, social and financial barriers that patients of LMICs face [11, 32]. These barriers lead to delayed presentation with advanced disease and poor compliance to treatment. The inability to complete the intended treatment leads to inferior outcomes as has been reported in the literature [33].

## Limitations

There are certain drawbacks of our study, the major being that it was a single institute, retrospective analysis of a small patient number and data on toxicities arising from the treatment were not clearly available due to the retrospective nature. However, ES is a rare disease and it is challenging to conduct a prospective randomised trial. Nevertheless, our study truly represents the real world data on epidemiology, clinical profile and outcomes of the patients of ES typically presenting in LMICs and the challenges faced in a resource-limited setting.

## Conclusion

This real world data from a resource-limited setting reports the delayed diagnosis of advanced disease and poor compliance to adjuvant consolidation chemotherapy in ES patients. This leads to significantly inferior outcomes when compared to outcomes from the west or India. Results of our analysis prompt the need for the development of dedicated multidisciplinary oncology centres in rural areas to remove the geographical barriers with a hub and spoke pattern with tertiary care centres. There is a dire need to initiate patient awareness programmes to overcome social barriers and improve financial support to these patients through public health funding to improve treatment compliance and outcomes.

## Conflicts of interest

There are no conflicts of interest to disclose.

## Funding

None.

## Author contributions

The manuscript has been read and approved by the authors and all have contributed to it.

Dr Nidhi Gupta: concept, data collection, data analysis, preparation and finalization of the draft.

Dr Kislay Dimri: supervision of data collection and revision of draft.

Dr Sudhir Kumar Garg: concept and revision of draft.

Dr Aanchal Arora: Data Collection and data analysis.

Dr Awadhesh Pandey: concept and supervision of data collection.

## Figures and Tables

**Table 1.** Demographic profile of ES patients at presentation.

Parameter	*n* = 81 (%)
Age (In years)	
0–10	12 (14.8)
11–20	39 (48.1)
21–30	25 (30.9)
>31	5 (6.2)
Median age	16 (3–61)
Sex	
Male	51 (63)
Female	30 (37)
Residence	
Urban	26 (32.1)
Rural	55 (67.9)
Marital status	
Single	65 (80.24)
Married	16 (19.7)
Morbidity	
Epilepsy	3 (3.7)
Tuberculosis	4 (4.9)
None	74 (91.3)
Addiction	
Smoking	4 (4.9)
Alcohol	3 (3.7)
Smoking and alcohol	3 (3.7)
None	71 (87.6)

**Table 2.** Clinicopathological profile of ES patients at presentation.

Parameter	*n* = 81 (%)
Presenting symptom	
Pain	58 (71.6)
Swelling	61 (75.3)
Restricted movement	19 (23.5)
History of trauma	10 (12.3)
Pathological fracture at presentation	6 (7.4)
Duration of symptoms before reporting	
3 months	21 (25.9)
4–6 months	36 (44.4)
7–12 months	18(22.2)
>12 months	6 (7.4)
Site	
Extremity	52 (64.2)
Pelvis	19 (23.5)
Axial skeleton/chest wall/face	4 (4.9)
Soft tissue/extraskeletal	6 (7.4)
Common extremity subsite	
Femur	17 (32.7)
Tibia	17 (32.7)
Humerus	6 (11.5)
Scapula/distal clavicle	4 (7.7)
Laterality	
Left	40 (49.4)
Right	41 (50.6)
Radiological investigation for primary	
MRI	73 (90.1)
CT scan	8 (9.9)
Radiological size of primary	
Less than 8 cm	30 (37)
More than or equal to 8 cm	51 (63)
Radiology consistent with ES	35 (43.2)
Disease at presentation	
Localised	63 (77.8)
Metastatic	18 (22.2)
Bone marrow positive	7 (8.6)

ES, Ewing sarcoma; MRI, Magnetic resonance imaging; CT, Computed tomography

**Table 3.** Treatment for LD (*n* = 58).

Parameter	*n* (%)
**NACT**	**58 (100)**
Median number of cycles	5 (2–12)
VAC	5 (8.6)
VAC/IE	53 (91.3)
**Local treatment**	**50 (86.2)**
**Surgery**	**28**
Limb salvage surgery	22
Amputation	6
Margin positive	3
**Radiotherapy**	**32**
**Definitive radiotherapy**	**22**
Postoperative radiotherapy (Surgery +RT)	4
Preoperative radiotherapy	6
Dose: 45 Gy	5
Dose: 50–54 Gy	11
Dose: 55–60 Gy	16
**Adjuvant chemotherapy**	**41 (70.7)**
Median number of cycles	7 (1-15)
VAC	2 (4.9)
VAC/IE	39 (95.1)
**Haematological toxicity**	
Anemia	5 (8.6)
Neutropenia	12 (20.7)
Neutropenic sepsis and shock	1 (1.7)
**Progressive disease on/4 weeks after adjuvant chemotherapy**	**16 (39.1)**

LD, Localized disease; NACT, Neoadjuvant chemotherapy; VAC, Vincristine/Adriamycin/Cyclophosphamide; IE, Ifosfamide/Etoposide; RT, Radiotherapy; Gy, Gray

**Table 4.** Treatment for relapse/progressive/MD.

Parameter	*n* (%)
**Treatment for relapse/progressive disease (*n* = 29)**	
**Site of recurrence/progression**	
Bone	8 (27.6)
Lungs	11 (37.9)
Lungs and bones	6 (20.7)
Lungs, bone and liver	2 (6.9)
Local site	2 (6.9)
**Treatment**	
**Chemotherapy**	**16 (55.2)**
Median number of cycles	2 (1–6)
Gemcitabine/Docetaxel	9
Cisplatin/adriamycin	2
VAC or IE or VAC/IE	5
Pazopanib	1
**Surgery**	**3 (10.3)**
Amputation	2
Local resection	1
**Radiotherapy**	**5 (17.2)**
20–30 Gy	3
45–50 Gy	2
**Treatment for MD (*n* = 18)**	
**Sites of metastases**	10 (55.6)
Lungs	2 (11.1)
Bones	4 (22.2)
Lungs and bones	2 (11.1)
Lungs and lymph nodes	**16 (88.9)**
**First line chemotherapy**	11 (1–17)
Median number of cycles	5
VAC	11
VAC/IE	**4 (22.2)**
**Second line chemotherapy**	2 (1–4)
Median number of cycles	1
IE	1
Docetaxel + Gemcitabine	2
Pazopanib	**1 (5.6)**
**Surgery**	1
Amputation	1
**Radiotherapy**	**12 (66.7)**
Definitive (54–60 Gy)	6
Palliative (20–30 Gy)	5
Whole lung irradiation (12 Gy/7 fractions)	1

MD, Metastatic disease; VAC, Vincristine/Adriamycin/Cyclophosphamide; IE, Ifosfamide/Etoposide; Gy, Gray

**Table 5.** OS: univariate and multivariate analysis for patients with LD (*n* = 58).

Variable	Univariate analysis	Multivariate Analysis				
	**HR**	**CI**	***p-*value**	**HR**	**CI**	***p* value**
Age >15 years	1.11	(0.57–2.32)	0.76			
Duration of Presenting symptoms >6 months	0.88	(0.39–1.96)	0.75			
Primary site (Pelvis versus Extremity)	1.05	(0.64–1.73)	0.81			
Primary tumor Size >8 cm	0.31	(0.13–0.73)	**0.008**	0.39	(0.16–0.95)	**0.04**
Pathological fracture	2.4	(0.33–18.3)	0.37			
Number of chemotherapy cycles >/= 15	0.24	(0.09–0.65)	**0.005**	0.25	(0.09–0.7)	**0.01**
Surgery as local treatment	0.56	(0.12–2.5)	0.45			
Local versus MD	3.87	(2.11–7.09)	**0.001**			

OS, Overall survival; LD, Localized disease; HR, Hazard ratio; CI, Confidence interval; MD, Metastatic disease
